# Ablating Tau Reduces Hyperexcitability and Moderates Electroencephalographic Slowing in Transgenic Mice Expressing A53T Human α-Synuclein

**DOI:** 10.3389/fneur.2020.00563

**Published:** 2020-06-19

**Authors:** Samuel T. Peters, Allyssa Fahrenkopf, Jessica M. Choquette, Scott C. Vermilyea, Michael K. Lee, Keith Vossel

**Affiliations:** ^1^Department of Neurology, N. Bud Grossman Center for Memory Research and Care, University of Minnesota, Minneapolis, MN, United States; ^2^Department of Neuroscience, University of Minnesota, Minneapolis, MN, United States; ^3^Institute for Translational Neuroscience, University of Minnesota, Minneapolis, MN, United States; ^4^Geriatric Research Education and Clinical Center, Minneapolis Veterans Affairs Health Care System, University of Minnesota, Minneapolis, MN, United States

**Keywords:** tau, α-synuclein, dementia with Lewy bodies, Parkinson's disease, hyperexcitability, EEG, sleep

## Abstract

Abnormal intraneuronal accumulation of the presynaptic protein α-synuclein (α-syn) is implicated in the etiology of dementia with Lewy bodies (DLB) and Parkinson's disease with dementia (PDD). Recent work revealed that mice expressing human α-syn with the alanine-53-threonine (A53T) mutation have a similar phenotype to the human condition, exhibiting long-term potentiation deficits, learning and memory deficits, and inhibitory hippocampal remodeling, all of which were reversed by genetic ablation of microtubule-associated protein tau. Significantly, memory deficits were associated with histological signs of network hyperactivity/seizures. Electrophysiological abnormalities are often seen in parkinsonian dementias. Baseline electroencephalogram (EEG) slowing is used as a supportive diagnostic feature in DLB and PDD, and patients with these diseases may exhibit indicators of broad network dysfunction such as sleep dysregulation, myoclonus, and seizures. Given the translational significance, we examined whether human A53T α-syn expressing mice exhibit endogenous-tau-dependent EEG abnormalities, as measured with epidural electrodes over the frontal and parietal cortices. Using template-based waveform sorting, we determined that A53T mice have significantly high numbers of epileptiform events as early as 3–4 months of age and throughout life, and this effect is markedly attenuated in the absence of tau. Epileptic myoclonus occurred in half of A53T mice and was markedly reduced by tau ablation. In spectral analysis, tau ablation partially reduced EEG slowing in 6–7 month transgenic mice. We found abnormal sleeping patterns in transgenic mice that were more pronounced in older groups, but did not find evidence that this was influenced by tau genotype. Together, these data support the notion that tau facilitates A53T α-syn-induced hyperexcitability that both precedes and coincides with associated synaptic, cognitive, and behavioral effects. Tau also contributes to some aspects of EEG slowing in A53T mice. Importantly, our work supports tau-based approaches as an effective early intervention in α-synucleinopathies to treat aberrant network activity.

## Introduction

Aberrant network excitability has come to the forefront as a significant contributor to cognitive decline in neurodegenerative disorders. Rates of seizures in dementia with Lewy bodies (DLB) and Alzheimer's disease (AD) are estimated to be ten-fold higher than in age-matched controls, and seizures in AD are associated with faster cognitive decline (1–3). Extended neurophysiological monitoring has revealed that ~40% of AD patients exhibit epileptiform activity even in the absence of seizures, and this subclinical phenomenon could accelerate disease progression (4). Additionally, myoclonus, another indicator of cortical hyperexcitability that often appears in DLB and cases of familial parkinsonism with dementia, may hasten disease onset (1, 5–7). Animal models recapitulate these findings. Transgenic mice modeling the amyloid-beta (Aβ) pathology of AD display both ictal and interictal epileptiform activity, as well as an increased susceptibility to chemically-induced seizures (8, 9). In the J20 transgenic mouse model, which overexpresses human amyloid precursor protein (hAPP) with familial-AD-associated mutations that result in high brain levels of Aβ, treatment with anti-seizure drugs not only reduced epileptiform activity, but also ameliorated synaptic deficits, cognitive dysfunction, and learning and memory impairment (10). Likewise, transgenic mice modeling Lewy body pathology have been shown to exhibit generalized tonic-clonic seizures, myoclonic burst seizures, and interictal epileptiform activity (11). Findings in AD have contributed to significant progress in this field of research, leading to clinical trials. The role of seizures and epileptiform activity in parkinsonian dementias, however, remains less well-studied.

Other electroencephalographic (EEG) abnormalities are present in DLB, as well as clinically similar Parkinson's disease with dementia (PDD). A majority of DLB cases present with dominant frequencies below the alpha range, such that slow-wave activity has been established as a supportive feature in DLB diagnosis (12–14). In both humans and transgenic mouse models of DLB/PDD pathology, this slowing of background activity is seen predominantly in the parietal cortex (11). Though the mechanisms of diffuse slowing are not well understood, some evidence indicates that inhibition of synaptic vesicle release has a role in the spectral shift from high-frequency oscillations (gamma, beta, alpha) toward lower frequencies (delta, theta) (15, 16). The presynaptic protein α-synuclein (α-syn), a central component of the fibrillar Lewy cytoplasmic and neuritic inclusions in DLB and PDD, has been shown to modulate neurotransmission (17, 18). Mutations in *SNCA*, the gene encoding α-syn, as well as genomic multiplication of *SNCA*, result in autosomal dominant parkinsonism (6, 19–23). Of particular interest is the alanine-53-threonine point mutation (A53T), which has been linked to postsynaptic dysfunction and tau pathology in addition to the presynaptic pathology associated with wild-type α-syn overexpression (24–26).

Recent work has indicated that the microtubule-associated protein tau is an essential facilitator of A53T α-syn-induced deficits in mice, including long-term potentiation (LTP) deficits, impaired spatial learning and memory, and inhibitory hippocampal remodeling (27). Histological analysis of transgenic mice expressing human A53T α-syn revealed histological indicators of seizures, including fewer c-Fos expressing cells and reduced calbindin levels in the dentate gyrus of the hippocampus. Genetic ablation of tau attenuated these changes (28–30). In this study, we examined the role of tau in facilitating EEG abnormalities associated with A53T α-syn. In doing so, we provide support for the concept that targeting tau to treat chronic network hyperexcitability may be an effective early therapeutic strategy to combat α-syn-associated dementia.

## Materials and Methods

### Animals

In this study, we used four genotypes of mice, which were bred and maintained as previously described (25, 27). Briefly, A53T mice (Jackson Labs, #006823) are heterozygous carriers of a mutant human α-syn transgene under control of the mouse prion promoter (MoPrP). In the mouse tau knockout line (mTau^−/−^, Jackson Labs #004779), the *Mapt* gene encoding endogenous mouse tau is disrupted by an EGFP coding sequence in the first exon, ablating tau's expression (31). mTau^−/−^ mice were bred with A53T mice to produce a line that harbors the mutant transgene and lacks endogenous tau. Nontransgenic (NTG) littermates of A53T mice served as controls. All mice were maintained in the C57BL/6J background strain. Animals were housed in a specific pathogen-free barrier facility with a 14-h light/10-h dark cycle and *ad libitum* access to food and water. All experiments were approved by the Institutional Animal Care and Use Committee of the University of Minnesota.

### EEG Surgeries

Approximately equal numbers of male and female mice were implanted for EEG and EMG recording (Supplementary Table 1). 1.2 mg/kg subcutaneous buprenorphine was used as an analgesic. To anesthetize mice, 400 mg/kg of 2,2,2-tribromoethanol (Sigma, St. Louis, MO, USA) was delivered via intraperitoneal injection. When necessary, the anesthetic regimen was supplemented with 1–2% isoflurane and 0.5–0.8 L/min O_2_. The scalp was shaved, the head was secured in a stereotaxic mount, and ophthalmic lubricant was applied to the eyes. The scalp was scrubbed with povidone-iodine antiseptic, and a ~1 cm rostral-caudal incision was made in the scalp to expose bregma. Four burr holes were made for epidural screw electrode placement, with two on each side of the skull placed symmetrically across the midline (two at 1 mm anterior/posterior and 1 mm medial/lateral; two at−2 mm anterior/posterior and 2 mm medial/lateral relative to bregma). 1-mm diameter epidural screws with pre-soldered wires were secured in the burr holes and insulated with dental acrylic. An 8-pin connector plug was soldered to the leads such that the screws posterior to bregma would record on separate parietal channels with reference to the left anterior screw (#8431-SM, Pinnacle Technology, Lawrence, KS, USA). The right anterior screw was used as ground. To aid sleep analysis, some mice had two stainless steel EMG leads embedded behind the skull in the nuchal muscle, separated by about 5 mm, and sutured into place. Exposed wiring was covered with dental acrylic. Mice recovered for at least 5–7 days before recording sessions.

### Video-EEG/EMG Recordings

Video-EEG/EMG was recorded with Sirenia Acquisition software (Pinnacle). Three different age groups were analyzed: 3–4 months (*n*_NTG_ = 10, *n*_mTau−/−_ = 10, *n*_A53T_ = 11, *n*_A53T/mTau−/−_ = 10), 6–7 months (*n*_NTG_ = 9, *n*_mTau−/−_ = 8, *n*_A53T_ = 10, *n*_A53T/mTau−/−_ = 10), and 9–10 months of age (*n*_NTG_ = 12, *n*_mTau−/−_ = 12, *n*_A53T_ = 9, *n*_A53T/mTau−/−_ = 9). Mice in the 3–4 month group were also recorded at 6–7 months, but those in the 9–10 month group were recorded only once. Some mice had dislodged or damaged electrodes after the first recording and could not be recorded again (1 NTG, 2 mTau^−/−^, 1 A53T, 4 A53T/mTau^−/−^). Four A53T/mTau^−/−^ mice that could not be recorded after 3–4 months were replaced with four that had not been recorded earlier for 6–7 month recordings. Each age group was analyzed separately.

Mice were tethered to a swivel commutator by a multichannel, 10x gain preamplifier during recording, which conducted biological signal to the data conditioning and acquisition system (Pinnacle). Mice were housed in clear cylindrical enclosures (10 in. diameter x 12 in. height) with fine-grain Sani-Chips bedding (P.J. Murphy Forest Products, Montville, NJ, USA) and given *ad libitum* access to food and water. The light/dark cycle in the recording room was identical to that of the housing room. Traces from the left and right parietal cortices were recorded on separate channels for ~24 h at a sampling rate of 2,000 Hz. High- and low-pass analog filters were applied with settings of 0.5 and 200 Hz for EEG signal and 10 and 1000 Hz for EMG signal. Synchronized video accompanied data acquisition. Traces were exported into European Data Format. All analyses were conducted while blinded to genotype.

### Data Analysis

#### Detection of Epileptiform Events and Seizures

Epileptiform events were defined as transient (≤ 75 ms) high-amplitude (>5 z-scores from the baseline mean) deflections arising suddenly from the background. Spikes were detected using a template-based, semi-automated approach in Spike2 software (Cambridge Electronic Design, Cambridge, UK) (8). Briefly, 200 ms of candidate waveforms were extracted from bilateral traces to a stereotrode channel, or a monotrode channel if signal from one electrode was severely distorted, and plotted into principal component (PC) space. K means clustering (K = 10 to 20) segregated the waveforms into groups based on similar component variances. The centroids of these groups formed templates to which all spikes were compared and reassigned based on shape. Waveforms matching artifact templates were excluded from subsequent clustering. The PC/K means refinement process was reiterated several times to distinguish large epileptiform events from artifacts or other high-amplitude features such as cortical sleep spindles. Recordings in which PC analysis (PCA) was insufficient to remove artifacts had all questionable event templates manually evaluated. Common artifacts were identified by manual video review and were often caused by chewing, scratching, or grooming (Supplementary Figure 1).

Epileptic myoclonic bursting was defined as cortical spikes occurring with sudden simultaneous muscle spasms such as neck jerking, forelimb clonus, rearing/falling, tail extension, and momentary loss of righting reflex. Presence or absence of myoclonic bursting in transgenic mice of each age was identified by manual video review. Generalized tonic-clonic seizures (GTCS) differed behaviorally from myoclonic bursting in that they involved continuous convulsions of fore- and hind limbs (>15 s) with sustained loss of righting reflex. Seizures were detected electroencephalographically using an amplitude setting (>7 z-scores from the baseline mean for at least 3 s) in Seizure Pro software (Pinnacle) and consisted of high-amplitude continuous polyspikes arising from the background. Detected seizures were confirmed by video review. One A53T/mTau^−/−^ mouse was excluded from event detection, myoclonus, and seizure analysis in the 9–10 month group due to severe distortion of EEG signal for significant portions of the recording, which interfered with templating.

#### Spectral Power

To analyze changes in baseline frequency, video data from daytime recordings was reviewed to identify 20- to 60-s periods of resting wakefulness in the absence of grooming, eating, or other behaviors or artifacts that artificially alter EEG signal. Using Spike2 software, raw signal data underwent 4,096-point Hanning fast Fourier transform, yielding a resolution of ~0.5 Hz. A 60-Hz digital notch filter was applied in all cases in which an electrical artifact was observed. 0.5-Hz bins were normalized as a percent of total spectral power in the left parietal channel between 0.5 and 50 Hz. The cumulative distribution function (CDF) of each genotype was calculated using mean integrals of data, and frequency bands were grouped as follows: delta = 0.5–4 Hz; theta = 4–10 Hz; alpha = 10–13 Hz; beta = 13–20 Hz; gamma = 20–50 Hz (11). Mice in which at least 20 s of artifact-free resting wakefulness could not be obtained, as determined by video-EEG review, were excluded (3–4 months: 2 A53T, 1 A53T/mTau-/-; 6–7 months: 1 A53T; 9–10 months: 2 NTG).

#### Sleep Scoring

Previous studies have shown that EEG and EMG recordings can be reliably used to assess vigilance states in mice, i.e. wakefulness, rapid eye movement (REM) sleep, and non-REM (NREM) sleep (32–34). It is well-established that mice are nocturnal and exhibit circadian intervals of activity and rest cycles, with a majority of their sleep occurring during the light period. In the current study, the two phases are referred to as the active phase (dark cycle) and quiescent phase (light cycle). A 24-h period, consisting of 14-h light and 10-h dark, was semi-automatically scored using Sirenia Sleep Pro (Pinnacle). 10-second epochs were scored as wake, NREM sleep, or REM sleep. Each state was determined by a specific combination of EEG and EMG patterns. Wakefulness was characterized by baseline level EEG oscillations, as determined by video review of wakeful states, with mixed frequency components between delta and gamma, and paired with greater than three-fold EMG power increases from motionless amplitude. This was coupled with video confirmation of movement. NREM sleep was characterized by a three-fold power increase in EEG amplitude from baseline, predominantly consisting of delta frequencies, paired with no EMG activity. REM sleep was characterized by baseline EEG amplitude comprised mainly of theta waves paired with no EMG activity except occasional muscle twitching. Four mice in the 6–7 month cohort and all mice at 9–10 months were manually scored relying solely on EEG and video recordings to monitor movement due to low quality or missing EMG data. This method has been verified in past studies as a reliable means to measure sleep/wake activity (8). Some mice, however, had to be excluded for the following reasons: file corruption that eliminated video feed (3–4 months: 1 A53T); severe distortion of EEG signal for the majority of the recording (9–10 months: 1 A53T/mTau-/-); and an inability to consistently distinguish between wakefulness and REM sleep or slow waves and NREM sleep for the full duration of the recording period, which was more common in 9–10 month old mice, for which scoring was done without the aid of EMG (3–4 months: 1 A53T/mTau-/-; 6–7 months: 1 NTG, 1 A53T, 1 A53T/mTau-/-; 9–10 months: 2 NTG, 1 mTau-/-, 2 A53T, 3 A53T/mTau-/-). Sleep/wake patterns were determined by calculating the total duration of each vigilance state over each period. Sleep onset latency was determined as the latency from wakefulness to non-REM sleep from the beginning of the morning quiescent (lighted room) phase. All data are shown relative to 14 h in the quiescent phase and 10 h in the active phase.

### Statistical Analysis

All statistical analyses were carried out in Prism version 8 (GraphPad Software, San Diego, CA, USA). Genotypic differences in epileptiform spike counts were assessed by nonparametric Kruskal-Wallis ANOVA with Dunn's *post hoc* test to analyze the effect of genotype on epileptiform event frequency. The Kruskal-Wallis ANOVA reports differences between groups with non-Gaussian distribution or low sample size and was applied for analyses that failed tests for normality. For frequency band data, statistical significance was determined by a repeated measures mixed model followed by Holm-Sidak correction, with genotype and frequency band factors. Frequency band served as the time axis for repeated measures. Only the genotype^*^frequency band interaction is reported, as the differences between genotypes in each frequency band are the measure of interest. To detect more subtle overall differences in baseline frequencies between groups, the CDF was calculated as the integral of the mean normalized distribution of each group. The CDF of each genotype was compared by the Kolmogorov-Smirnov test, which calculates the probability of a difference in the distributions based on the maximum distance between CDFs. Results from CDF comparisons were corrected for multiple comparisons using Holm-Sidak correction. Sleep data were analyzed using a one-way ANOVA, with genotype as the independent variable, and multiple comparisons were analyzed with the Tukey test. The null hypothesis was rejected at *p* < 0.05. Results are reported as nominal p-values that retained significance after correction for multiple comparisons.

## Results

### Tau Ablation Attenuates Epileptiform Activity in A53T Mice

**Figure 1:**
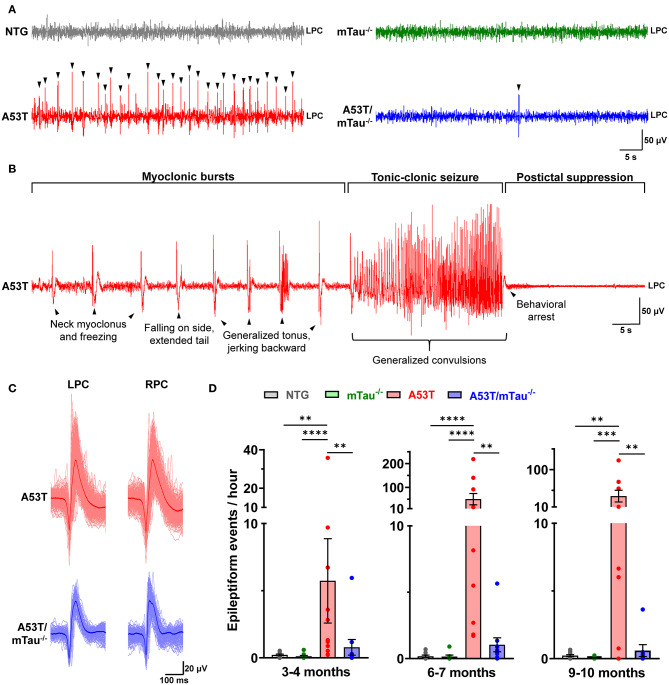
Knockout of endogenous tau reduces cortical epileptiform events in A53T mice. **(A)** Representative traces from 6 to 7 month old nontransgenic (NTG), mTau^−/−^, A53T, and A53T/mTau^−/−^ mice during NREM sleep. Arrowheads indicate epileptiform events. **(B)** A generalized tonic-clonic seizure in an A53T mouse aged 6.2 months. The seizure was preceded by neck and forelimb myoclonus, lasted ~30 s with loss of righting reflex, and was followed by postictal dampening of signal and behavioral arrest. **(C)** The average waveform of events initially crossing the negative threshold from the A53T and A53T/mTau^−/−^ mice in **(A)**. Epileptiform complexes were characterized by a cross-channel, high-amplitude spike, and were usually followed by slow waves. Aligned events contributing to the average are overdrawn (light red for A53T, light blue for A53T/mTau^−/−^). **(D)** Epileptiform events detected in mice in the 3–4 month group [Kuskal-Wallis ANOVA, $\chi_{\left(3\right)}^{2}$ = 18.51, *p* = 0.0003], 6–7 month group [$\chi_{\left(3\right)}^{2}$ = 23.85, *p* < 0.0001], and 9–10 month group [$\chi_{\left(3\right)}^{2}$ = 15.72, *p* = 0.0013]. At each age, events were detected more frequently in A53T mice than any other group. Events were rare in control groups. Kruskal-Wallis nonparametric ANOVA was followed with Dunn's *post hoc* test. 3–4 months: *n*_NTG_ = 10, *n*_mTau−/−_ = 10, *n*_A53T_ = 11, *n*_A53T/mTau−/−_ = 10; 6–7 months: *n*_NTG_ = 9, *n*_mTau−/−_ = 8, *n*_A53T_ = 10, *n*_A53T/mTau−/−_ = 10; 9–10 months: *n*_NTG_ = 12, *n*_mTau−/−_ = 12, *n*_A53T_ = 9, *n*_A53T/mTau−/−_ = 8. **p < 0.01, ***p < 0.001, ****p < 0.0001. Bars represent means ± SEM. LPC, left parietal cortex; RPC, right parietal cortex.

After review of several EEG traces from mice of each genotype, we found epileptiform events occurring in A53T and A53T/mTau^−/−^ mice and rare generalized seizures in A53T mice (Figures 1A,B). NTG and mTau^−/−^ controls exhibited normal background activity without appreciable epileptiform activity (Figure 1A). Epileptiform events usually consisted of single or bidirectional spikes <75 ms in width, and were often followed by >100-ms slow waves before returning to baseline. These features were identifiable bilaterally, and the initial spike deflection was most often negative relative to the reference (Figure 1C). Semi-automated quantitation of events by iterative PC clustering and sorting was effective at detecting prominent spikes throughout 24-h recordings. Manual verification of several recordings demonstrated an average specificity of ~93% and an average sensitivity of ~75% (Supplementary Figure 1). Using this method, we determined that epileptiform events occur in A53T mice in each age group (Figure 1D). Blinded quantitation confirmed that these events seldom occur in either control group. Kruskal-Wallis ANOVA using genotype as the independent variable determined that genotype impacts the distribution of epileptiform event frequency in all age groups. In each group, A53T mice had significantly higher numbers of these events than controls, and this presentation was attenuated by tau ablation (Figure 1D). This was particularly notable in the 6–7 and 9–10 month groups, where the mean event frequencies in A53T/mTau^−/−^ were reduced ~50- to 60-fold (6–7 months: A53T = 50.33/h, A53T/mTau^−/−^ = 1.04/h; 9–10 months: A53T = 36.17/h, A53T/mTau^−/−^ = 0.59/h).

Isolated epileptiform events occasionally occurred with myoclonus, indicating epileptic myoclonus due to cortical hyperexcitability (1, 2, 35). Epileptic myoclonus was detected in 50% of all A53T mice across ages, while only one A53T/mTau^−/−^ mouse out of 17 exhibited epileptic myoclonus at any age (Table 1). Generalized tonic-clonic seizures (GTCSs) were detected twice in different A53T mice over the course of the 24 h recordings – one at 6 months (Supplementary Video 1) and the other at 10 months (Supplementary Video 2). GTCSs lasted ~20–30 s and were accompanied by fore- and hind limb convulsions, momentary loss of righting reflex, and tail extension. In both mice, GTCSs were preceded by periodic myoclonus and followed by a sustained period of EEG suppression (Figure 1B). We did not find GTCSs in control or A53T/mTau^−/−^ mice.

**Table 1:** Prevalence of epileptic myoclonus and generalized tonic-clonic seizures in mice expressing A53T α-synuclein with or without endogenous tau.

**Age (months)**	**Epileptic myoclonus**		**Generalized tonic-clonic seizures**	
	**A53T**	**A53T/mTau^**−/−**^**	**A53T**	**A53T/mTau^**−/−**^**
3–4	2/10 (20%)^1^	0/10 (0%)	0/10 (0%)^1^	0/10 (0%)
6–7	3/10 (30%)	0/10 (0%)	1/10 (10%)	0/10 (0%)
9–10	5/9 (56%)	1/8 (13%)^2^	1/9 (11%)	0/8 (0%)^2^
Lifetime	9/18 (50%)^*^	1/17 (6%)^*^	2/18 (12%)	0/17 (0%)

1 *One excluded due to video failure*.

2 *One excluded due to severe distortion of EEG*.

* *p = 0.007 (Fisher exact test, A53T vs. A53T/mTau^−/−^)*.

### EEG Slowing in A53T Mice Is Partially Reduced by Tau Knockout

**Figure 2:**
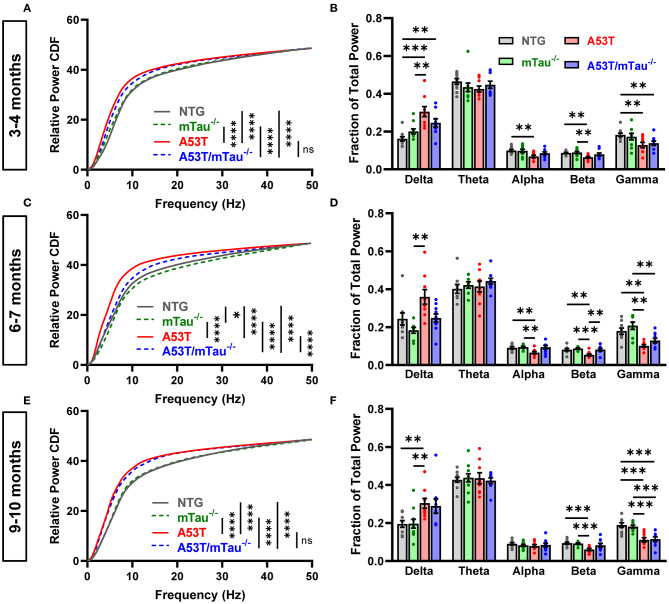
Tau ablation partially ameliorates EEG slowing in A53T mice during resting wakefulness. We used two different methods to assess the effect of genotype on EEG frequencies during 20–60 s of quiet wakefulness in different age groups. To compare the cumulative distributions of genotypes at each age, pairwise Kolmogorov-Smirnov tests were done, each yielding a distance statistic, *D*. The derived p-values were sorted using Holm-Sidak correction for multiple comparisons. **(A)** Cumulative frequency distributions in the 3–4 month age group. Transgenic genotypes were left-shifted from controls. Nontransgenic (NTG) vs. mTau^−/−^ (*D* = 0.1359, p = 0.297), NTG vs. A53T (*D* = 0.3495, p < 0.0001), NTG vs. A53T/mTau^−/−^ (*D* = 0.3398, *p* < 0.0001), mTau^−/−^ vs A53T (*D* = 0.3592, *p* < 0.0001), mTau^−/−^ vs A53T mTau^−/−^ (*D* = 0.3301, *p* < 0.0001), A53T vs. A53T/mTau^−/−^ (*D* = 0.1553, p = 0.166). **(B)** Frequency band analysis using a repeated measures mixed-effects model revealed a significant interaction of genotype and frequency band. [*F*_(12, 170)_ = 6.709, *p* < 0.0001]. Holm-Sidak *post hoc* tests showed that delta power is increased in mice expressing A53T α-syn, while alpha, beta, and gamma power are reduced. **(C)** In the 6–7 month group, both A53T and A53T/mTau^−/−^ mice were left-shifted of controls, though A53T/mTau^−/−^ were shifted toward higher frequencies than A53T mice with endogenous tau. 6–7 months: NTG vs. mTau^−/−^ (*D* = 0.2233, *p* = 0.012), NTG vs. A53T (*D* = 0.466, *p* < 0.0001), NTG vs. A53T/mTau^−/−^ (*D* = 0.3689, *p* < 0.0001), mTau^−/−^ vs A53T (*D* = 0.5437, *p* < 0.0001), mTau^−/−^ vs A53T mTau^−/−^ (*D* = 0.5049, *p* < 0.0001), A53T vs. A53T/mTau^−/−^ (*D* = 0.3495, *p* < 0.0001). **(D)** Similar to younger mice, a mixed-effects model revealed that genotype interacts significantly with frequency band in mice aged 6–7 months [*F*_(12, 160)_ = 6.432, *p* < 0.0001, Holm-Sidak *post hoc* test]. Distinct from 3–4 month mice, however, A53T/mTau^−/−^ mice in the 6–7 month age group have significantly higher beta power than A53T mice. (**E**) At 9–10 months, the cumulative distributions of both transgenic groups are shifted toward slower frequencies from controls and are not distinguishable from each other. 9–10 months: NTG vs. mTau^−/−^ (*D* = 0.1165, *p* = 0.487), NTG vs. A53T (*D* = 0.4466, *p* < 0.0001), NTG vs. A53T/mTau^−/−^ (*D* = 0.466, *p* < 0.0001), mTau^−/−^ vs. A53T (*D* = 0.4854, *p* < 0.0001), mTau^−/−^ vs A53T mTau^−/−^ (*D* = 0.4757, *p* < 0.0001), A53T vs. A53T/mTau^−/−^ (*D* = 0.1262, *p* = 0.385). **(F)** Differences between the A53T genotypes and controls at 9–10 months are pronounced in delta, beta, and gamma frequencies. *F*_(12, 175)_ = 5.073, *p* < 0.0001. All repeated measures mixed models used Geisser-Greenhouse correction to account for sphericity. 3–4 months: *n*_NTG_ = 10, *n*_mTau−/−_ = 10, *n*_A53T_ = 9, *n*_A53T/mTau−/−_ = 9; 6–7 months: *n*_NTG_ = 9, *n*_mTau−/−_ = 8, *n*_A53T_ = 9, *n*_A53T/mTau−/−_ = 10; 9–10 months: *n*_NTG_ = 10, *n*_mTau−/−_ = 11, *n*_A53T_ = 9, *n*_A53T/mTau−/−_ = 9. *p < 0.05, **p < 0.01, ***p < 0.001, ****p < 0.0001. Bars represent means ± SEM. CDF, cumulative distribution function.

Because EEG slowing is a prominent feature of human α-synucleinopathies, as well as transgenic mice modeling such diseases (7, 11, 12, 36), we examined the distribution of dominant EEG frequencies during resting wakefulness in two ways. The CDF of relative spectral power shows general changes in baseline rhythm, while separation of relative power into different frequency bands provides better resolution of specific rhythms. At 3–4 months of age, pairwise Kolmogorov-Smirnov comparisons revealed that the CDFs of both A53T and A53T/mTau^−/−^ mice are shifted toward slower frequencies when compared with controls (Figure 2A). Binning of power into frequency bands demonstrated that relative delta power is significantly increased in A53T and A53T/mTau^−/−^ mice compared with NTG mice at this age. NTG mice also had higher alpha, beta, and gamma activity compared to A53T mice and higher gamma activity compared to A53T/mTau^−/−^ mice (Figure 2B). Ablation of tau did not significantly impact the relative distribution of power in any frequency band in 3–4 month old A53T mice.

In the 6–7 month group, A53T mice were shifted left of controls as well (Figure 2C). Interestingly, the CDF of A53T/mTau^−/−^ mice in this age group was significantly right-shifted toward higher frequencies compared to A53T, though it was left of control groups at this age. 6–7 month old A53T mice had significantly less alpha, beta, and gamma activity than nontransgenic mice. In this group, A53T/mTau^−/−^ mice had significantly higher beta activity than A53T mice as well (Figure 2D). Kolmogorov-Smirnov tests also showed a right-shift of tau knockout compared to nontransgenic mice, though this was not apparent in any particular frequency band (Figures 2C,D).

Distribution patterns in the 9–10 month group resemble those in the 3–4 month. The baseline frequency distribution of A53T/mTau^−/−^ mice in the older group did not differ from their tau-expressing counterparts, and both groups showed slower CDFs than either control group (Figure 2E). Consistent with patterns in the younger mice, 9–10 month old A53T mice also had significantly higher delta power and lower beta and gamma power than NTG and mTau^−/−^ mice, though not alpha (Figure 2F). A53T/mTau^−/−^ mice had reduced gamma power compared to controls in the 9–10 month old group. Across age groups, we did not find any differences in theta activity (Figures 2B,D,F).

### Abnormal Sleep in A53T Mice Is Independent of Tau Genotype

**Figure 3:**
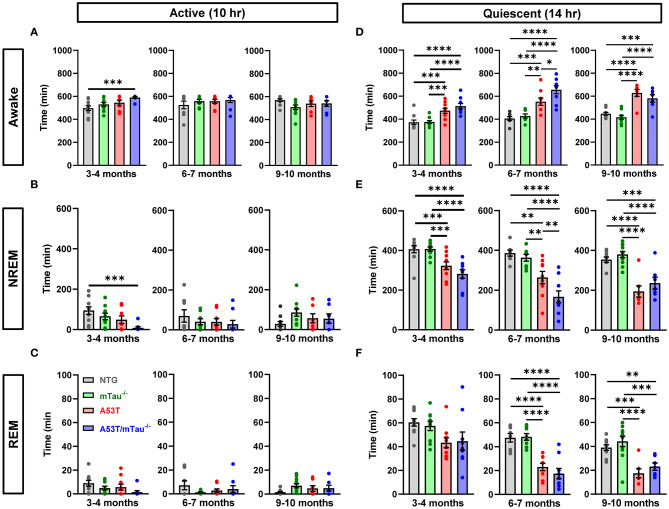
Abnormal sleep patterns in A53T mice are not dependent on tau. **(A)** During the active (dark) phase, all mice were awake for most of the 10-h cycle across age groups. One-way ANOVA determined that there are genotypic differences in time spent awake at 3–4 months. Tukey *post hoc* test demonstrated that A53T/mTau^−/−^ mice spent more time awake in the active phase than nontransgenic (NTG) mice. No differences were found in other genotype or ages. 3–4 months: *F*_(3, 35)_ = 4.642, *p* = 0.0078; 6–7 months: *F*_(3, 30)_ = 0.6481, *p* = 0.5903; 9–10 months: *F*_(3, 31)_ = 1.840, *p* = 0.1604. **(B)** Correspondingly, A53T/mTau^−/−^ mice had decreased NREM sleep in the active phase relative to NTG in the 3–4 month group. One-way ANOVA, 3–4 months: *F*_(3, 35)_ = 4.889, *p* = 0.0061; 6–7 months: *F*_(3, 30)_ = 0.7059, *p* = 0.556; 9–10 months: *F*_(3, 31)_ = 1.81, *p* = 0.1659. **(C)** The time spent in REM sleep during the active phase was low across all genotypes, and no significant differences were noted in any age group. 3–4 months: *F*_(3, 35)_ = 2.254, *p* = 0.0993; 6–7 months: *F*_(3, 30)_ = 0.9996, *p* = 0.4065; 9–10 months: *F*_(3, 31)_ = 1.841, *p* = 0.1603. **(D,E)** Mice generally spent more time sleeping in the quiescent (light) phase than the active phase. **(D)** Across age groups, genotype significantly impacted time spent awake in the quiescent phase [3–4 months: *F*_(3, 35)_ = 13.11, *p* < 0.0001; 6–7 months: *F*_(3, 30)_ = 17.78, *p* < 0.0001; 9–10 months: *F*_(3, 31)_ = 20.57, *p* < 0.0001]. After Tukey adjustment, A53T and A53T/mTau^−/−^ mice spent significantly more time awake than did controls, and A53T/mTau^−/−^ were awake for significantly more time on average than A53T at 6–7 months. **(E)** NREM sleep time in the quiescent phase was significantly impacted by the genotype factor in all age groups, and transgenic mice both with and without endogenous tau spent less time in NREM sleep than NTG or mTau^−/−^ mice during the light phase [3–4 months: *F*_(3, 35)_ = 12.03, *p* < 0.0001; 6–7 months: *F*_(3, 30)_ = 15.93, *p* < 0.0001; 9–10 months: *F*_(3, 31)_ = 18.89, *p* < 0.0001]. However, A53T/mTau^−/−^ mice accumulated less NREM sleep than A53T mice in the 6–7 month group. **(F)** ANOVA warranted further analysis of REM deficits in A53T mice at 3–4 months of age [*F*_(3, 35)_ = 3.245, *p* = 0.0334]. However, there were no significant differences after *post hoc* correction for multiple comparisons. The impact of genotype on REM deficits during the quiescent phase was evident in the older groups [6–7 months: *F*_(3, 30)_ = 20.49, *p* < 0.0001; 9–10 months: *F*_(3, 31)_ = 12.44, *p* < 0.0001]. All significant main effects in ANOVA were followed with the Tukey test. 3–4 months: *n*_NTG_ = 10, *n*_mTau−/−_ = 10, *n*_A53T_ = 10, *n*_A53T/mTau−/−_ = 9; 6–7 months: *n*_NTG_=8, *n*_mTau−/−_ = 8, *n*_A53T_ = 9, *n*_A53T/mTau−/−_ = 9; 9–10 months: *n*_NTG_ = 10, *n*_mTau−/−_ = 11, *n*_A53T_ = 7, *n*_A53T/mTau−/−_ = 7. *p < 0.05, **p < 0.01, ***p < 0.001, ****p < 0.0001. Bars represent means ± SEM.

Mice exhibited intervals of activity/rest cycles throughout 24 h, with the dark period being primarily characterized by states of wakefulness and the majority of sleep occurring in the light period. Analysis of 24-h periods consisting of 10 h of dark (active phase) and 14 h of light (quiescent phase) revealed abnormalities in the sleep/wake activity of A53T and A53T/mTau^−/−^ mice compared with controls in all age groups. All mice spent the majority of the 10-h active phase awake, though 3–4 month A53T/mTau^−/−^ mice spent more time awake and less time in NREM sleep than NTG mice during the active phase (Figures 3A,B). We did not discover any differences in total REM sleep during the active phase at any age (Figure 3C).

Sleep primarily occurred during the quiescent phase for all genotypes and ages. During this phase, both A53T and A53T/mTau^−/−^ mice spent more time awake and less time in NREM sleep than controls across age groups (Figures 3D,E). There were no notable differences in REM sleep in the 3–4 month group. However, in both of the older groups, total time in REM sleep was diminished in both A53T and A53T/mTau^−/−^mice (Figure 3F). There was no evidence suggesting that ablation of tau normalizes sleep patterns in mice that express A53T α-synuclein. In fact, 6–7 month A53T/mTau^−/−^mice had increased awake time and reduced time in NREM sleep compared to 6–7 month A53T mice.

**Figure 4:**
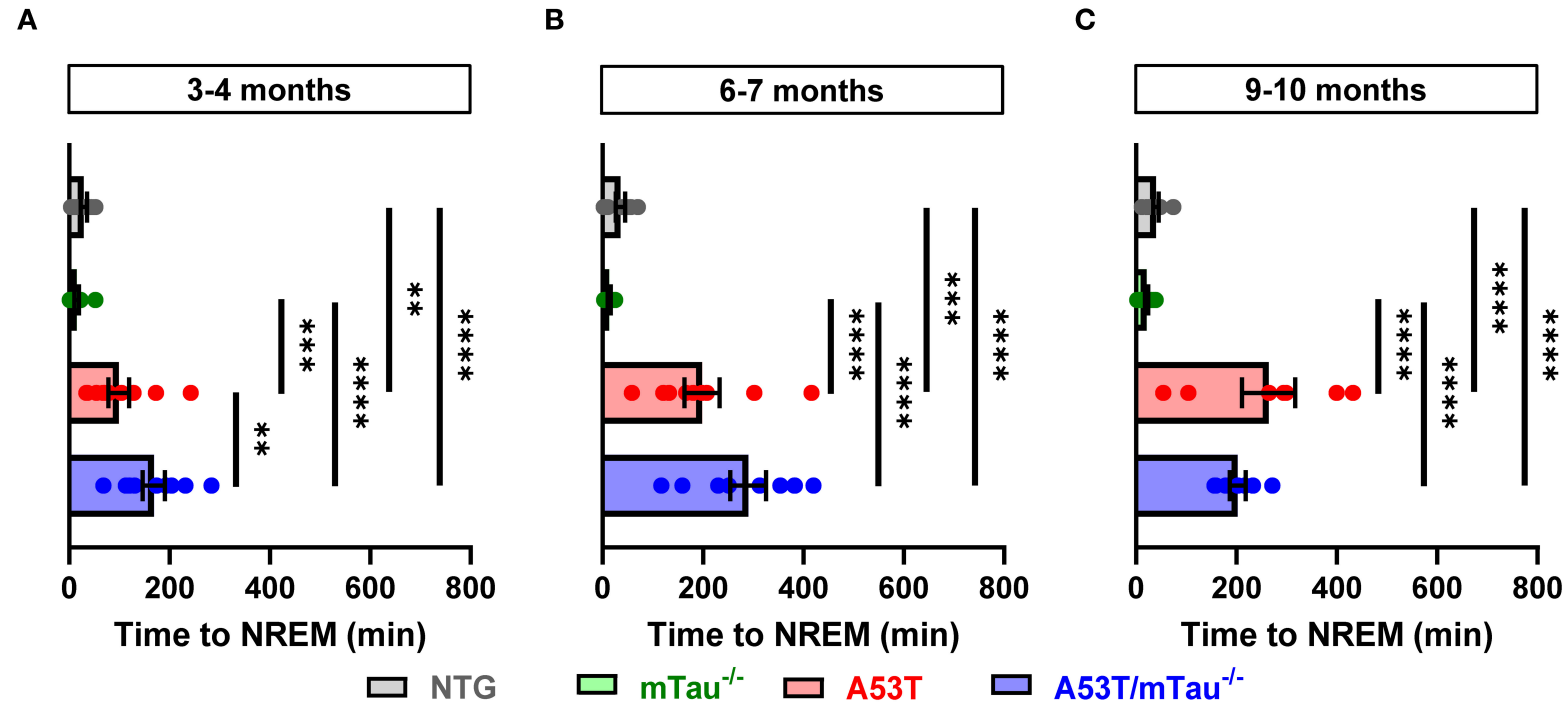
A53T and A53T/mTau^−/−^ mice have increased sleep onset latency periods from the end of the active phase. **(A–C)** Sleep onset latency was measured as the amount of time between the beginning of the morning quiescent phase and the first bout of NREM sleep. Across ages, all nontransgenic (NTG) and mTau^−/−^ mice fell asleep within the first 75 min. Genotype affected the duration of this latency period at each age as determined by one-way ANOVA [3–4 months: *F*_(3, 35)_ = 20.49, *p* < 0.0001; 6–7 months: *F*_(3, 30)_ = 24.15, *p* < 0.0001; 9–10 months: *F*_(3, 31)_ = 28.27, *p* < 0.0001]. A53T and A53T/mTau^−/−^ mice took significantly more time to reach NREM sleep than controls in all age groups. 3–4 months: *n*_NTG_ = 10, *n*_mTau−/−_ = 10, *n*_A53T_ = 10, *n*_A53T/mTau−/−_ = 9; 6–7 months: *n*_NTG_ = 8, *n*_mTau−/−_ = 8, *n*_A53T_ = 9, *n*_A53T/mTau−/−_ = 9; 9–10 months: *n*_NTG_ = 10, *n*_mTau−/−_ = 11, *n*_A53T_ = 7, *n*_A53T/mTau−/−_ = 7. **p < 0.01, ***p < 0.001, ****p < 0.0001. Bars represent means ± SEM.

In addition to reduced total sleep time, one-way ANOVA determined that latency to sleep during the quiescent phase is also significantly impacted by genotype. *Post hoc* analyses revealed that in all age groups, A53T and A53T/mTau^−/−^ groups both have longer latencies to sleep than either control group, in which all mice fell asleep within 75 min of the start of the quiescent phase (Figures 4A–C). In the 6–7 month group, A53T mice with ablated tau took longer to reach sleep than those with normal tau expression (Figure 4B). These data together suggest that although A53T α-synuclein expression is clearly a significant factor driving sleep dysregulation, tau is largely uninvolved in A53T-associated sleep deficits.

### A53T-Associated Epileptiform Events Occur Primarily Outside of REM Sleep

**Figure 5:**
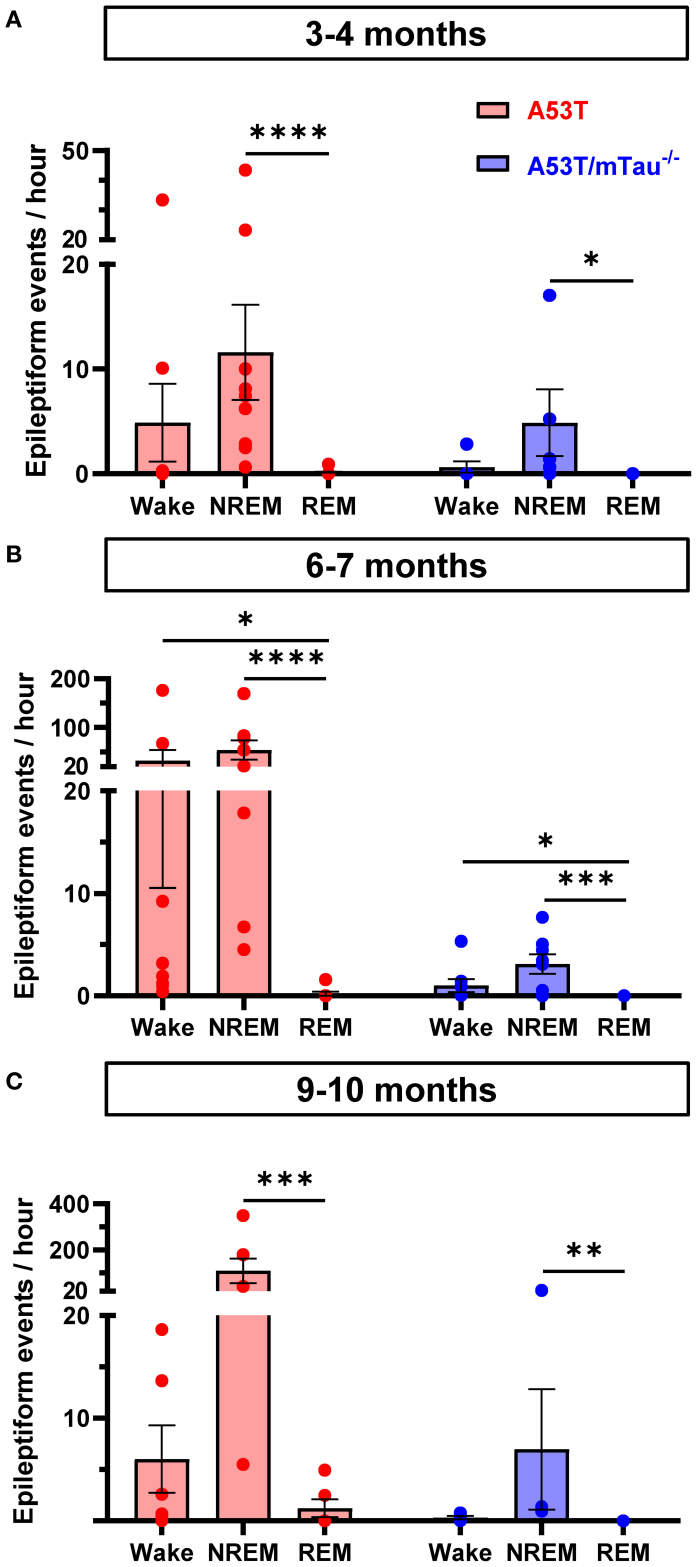
Epileptiform events in A53T and A53T/mTau^−/−^ mice occur primarily during NREM sleep and wakefulness. **(A)** Segregation of epileptiform events by vigilance state in the 3–4 month old group demonstrated that state of wakefulness significantly impacted frequency of epileptiform events [Kruskal-Wallis ANOVA, A53T: $\chi_{\left(2\right)}^{2}$ = 17.57, *p* = 0.0002; A53T/mTau^−/−^: $\chi_{\left(2\right)}^{2}$ = 7.462, *p* = 0.019]. Events were rarely detected in REM sleep in either A53T or A53T/mTau^−/−^ groups. **(B)** In the 6–7 month groups, a similar pattern was evident [A53T: $\chi_{\left(2\right)}^{2}$ = 18.27, *p* = 0.0001; A53T/mTau^−/−^: $\chi_{\left(2\right)}^{2}$ = 13.83, *p* = 0.001]. Mice in this age group had significantly more frequent epileptiform activity during wakefulness and NREM sleep than REM. **(C)** In the oldest age group, epileptiform activity during NREM sleep persisted [A53T: $\chi_{\left(2\right)}^{2}$ = 11.95, *p* = 0.0002; A53T/mTau^−/−^: $\chi_{\left(2\right)}^{2}$ = 10.2, *p* = 0.0002]. Significant main effects from Kruskal-Wallis ANOVA were followed with Dunn's *post hoc* test. Only mice with at least one detected epileptiform event and reliable sleep scoring data were included. 3–4 months: *n*_A53T_ = 10, *n*_A53T/mTau−/−_ = 6; 6–7 months: *n*_A53T_ = 9, *n*_A53T/mTau−/−_ = 8; 9–10 months: *n*_A53T_ = 6, *n*_A53T/mTau−/−_ = 4. *p < 0.05, **p < 0.01, ***p < 0.001, ****p < 0.0001. Error bars represent means ± SEM.

In 3–4, 6–7, and 9–10 month old mice, Kruskal-Wallis ANOVA using wakefulness state as the independent variable revealed that epileptiform event frequency is not uniform between stages of vigilance. Although A53T mice had markedly higher occurrence of epileptiform events than A53T/mTau^−/−^ mice (Figure 1C), the distribution pattern of these events across vigilance states was similar between groups at each age. Among mice with detected epileptiform activity, events in NREM sleep were more frequent than those in REM—which were relatively rare—across all age groups (Figures 5A–C). In the group aged 6–7 months, events occurred significantly more frequently during wakefulness in both genotypes as well. Some events occurring in periods of wakefulness coincided with myoclonic activity in A53T mice, but epileptic myoclonus was rarely observed in A53T/mTau^−/−^ mice (Table 1).

## Discussion

The results of this study indicate that endogenous tau is a critical mediator of network hyperexcitability in the A53T mouse model of α-synucleinopathy, even at young ages. Ablation of tau significantly reduces both frequency of epileptiform activity and incidence of epileptic myoclonus. Tau ablation also reduces EEG slowing associated with A53T α-synuclein expression in 6–7 month old mice. These findings, in conjunction with previous studies in A53T mice, suggest that tau-mediated aberrant network activity occurs early in pathogenesis, preceding and likely contributing to later deficits in synaptic function and behavior (25, 27).

Chronic hyperexcitability in neurodegenerative dementias is associated with earlier disease onset and faster cognitive decline (1–4, 35). The significance of tau in facilitating hyperexcitability has been well documented in mouse models of AD as well as models of epilepsy. Studies have shown that a loss of tau expression prevents early epileptiform activity and cognitive deficits in several models of AD (30, 37–39). Less, however, is known about the role of tau in mediating hyperexcitability in α-synucleinopathies. Previous work using 4–7 month old mice that express wild-type human α-syn driven by the Thy-1 promoter (line 61) demonstrated a partial reduction (~50%) in epileptiform activity and normalization of hippocampal biomarkers of hyperexcitability in transgenic mice lacking tau (11). Here, we show that tau ablation reduces epileptiform activity to levels similar to non-diseased controls (Figure 1D). The A53T α-syn model is unique from wild-type α-syn transgenic mouse models in its induction of tau-dependent postsynaptic deficits in addition to the presynaptic deficits associated with wild-type α-syn overexpression, suggesting additional gain-of-function effects of pathological tau in the A53T model (25, 27).

The mechanism by which tau facilitates hyperexcitability in this model is not well-understood. Tau missorting into dendritic spines, which occurs as a result of A53T α-syn expression, has been linked to calcineurin-dependent AMPA receptor (AMPAR) internalization (25, 40, 41). Significantly, we found epileptic activity occurring at earlier ages than AMPAR deficits occur in this model, suggesting that epileptic activity may be contributing to these deficits (27). A53T α-syn promotes these tau-dependent AMPAR deficits through dysregulation of glycogen synthase kinase-3β, which colocalizes with tau and A53T α-syn and contributes to parkinsonian pathology (42, 43). Though the biochemical and functional outcomes of hyperexcitability are becoming more apparent, their causal mechanisms have yet to be thoroughly studied in the context of α-synucleinopathies.

In the current study, it is likely that early network hyperactivity, as indicated by epileptiform events as early as 3–4 months, contributes to development of inhibitory hippocampal remodeling and synaptic and cognitive deficits in a tau-dependent manner in A53T mutant α-syn mice. Indeed, previous studies have shown that A53T mice exhibit hippocampal circuit remodeling, LTP deficits, and memory impairments starting around 6 months of age (27). The presence of epileptiform activity in A53T mice at 3–4 months of age—preceding the onset of cognitive deficits—provides further support that epileptiform activity is an early contributor to cognitive dysfunction. A53T mice begin to display a molecular signature associated with seizures in the hippocampus as early as 6 months, including reduction of c-Fos and calbindin in dentate granule cells, and increased ectopic neuropeptide Y expression (27). Even at 12 months, A53T mice lacking tau do not exhibit these changes. Although we recorded epileptiform activity in the cortex, evidence of inhibitory network remodeling in the hippocampus in the previous study further supports a mechanistic link between network hyperactivity and behavioral and memory deficits seen in the A53T mice. Delineating the propagation of network hyperactivity throughout the brain, as well as the regions involved in epileptogenesis, will be a necessary step on which future research should focus.

The contribution of EEG slowing to cognitive function in this model is unclear. Baseline and transient slowing in temporal and parietal regions is common in patients with DLB, and our data are consistent with previous work demonstrating parietal slowing in 4–8 month old mice overexpressing wild-type human α-syn (11, 14). The observation that ablation of tau reduces this shift in 6–7 month old A53T transgenic mice is novel, to the best of our knowledge, as the same phenomenon was not found to occur in the Thy-1 human α-syn mice of a wider age range. That spectral power in A53T/mTau^−/−^ mice was still left-shifted relative to controls at each age implies that tau may only be partially involved in this process. The slowing observed in this model, as well as Thy-1 driven wild-type α-syn mice, may be more attributed to pre-synaptic dysfunction, which is observed broadly in multiple transgenic α-syn mouse models. Modulation of synaptic transmission at the presynaptic level is a known function of α-syn (17), and previous work has demonstrated that pharmacological inhibition of glutamate transmission reduces the power of high-frequency cortical waves (15). Because tau ablation rescues cognitive function associated with the hippocampus but does not fully rescue EEG slowing, epileptiform activity may be more important to deficits in LTP and spatial learning and memory. It is possible, however, that this slowing is more pertinent to other higher-order cognitive functions. Increased delta activity and decreased alpha have been correlated with focused attention impairments in patients with mild cognitive impairment or AD (44). Diffuse EEG slowing has also been correlated with decreased cognitive speed in otherwise unimpaired older adults (45). Although learning and memory deficits in A53T mice depend on tau, the degree to which other cognitive deficits in this model are tau-dependent is unknown. Subsequent studies in different behavioral paradigms will shed light on the specific circuits in which A53T α-syn and tau interact.

The disruption of sleep/wake activity exhibited by A53T mice in the current study is in accordance with previous studies demonstrating that α-synucleinopathy affects normal rest-activity cycles (34). Similar to the current study, 9–10 month old Thy-1 human α-syn mice accumulated less REM sleep over a 24-h period than nontransgenic littermates. Notably, decreased REM sleep has been associated with Parkinson's disease in humans (46). Additionally, sleep alterations in A53T mice occur months before the deficits seen in motor function, consistent with the development of sleep disruptions in Parkinson's disease patients before the onset of motor symptoms. Thy-1 human α-syn mice demonstrated a significant increase in non-REM sleep during the quiescent phase, whereas the A53T mice in the current study exhibited a decrease in non-REM sleep during this phase. Complementary to our study, there is evidence that the overexpression of the A53T mutation of human α-syn under the Thy-1 promotor increased activity and decreased total sleep time in transgenic mice (47). However, the vigilance state in which these effects were observed was not quantitatively assessed. This suggests that distinctive mechanisms may be affecting circadian rhythms in the different α-syn models. Similar to the effects seen in the mice with the A53T mutation, it is well-established that many patients with Parkinson's disease experience an overall decrease in total sleep time compared to normal subjects (48). The sleep disruptions seen in the current study appear to be tau-independent, as ablation of tau did not eliminate the sleep disruptions observed in A53T mice. One possible explanation for this is that the observed sleep disruptions are due to dysfunction of brainstem regions that also regulate spontaneous locomotor activity, which is increased in A53T mice and not influenced by tau ablation (27, 49–51).

Epileptiform activity in AD patients occurs primarily during sleep, and particularly NREM sleep. Evidence from the Tg2576 mouse model of AD also indicates that spikes occur during REM sleep at 5 weeks of age and both REM and NREM sleep by 7 months of age (8). While REM sleep may inhibit spikes in humans, this process may become altered early in dementia (52, 53). As we observed epileptiform events to be elevated during NREM sleep in A53T mice, it will be important to include extended EEG to capture a sufficient sampling of all vigilance states in future human investigations of silent EEG abnormalities in α-synucleinopathies.

The present study was limited in its ability to establish a clear effect of age on progression of the hyperexcitable phenotype in A53T mice, as our younger and older cohorts consisted of different animals. The number of mice with multiple recordings at 3–4 and 6–7 months was limited as well, as many animals exhibited diminished EEG signal quality with age or dislodged the implanted electrodes. Moreover, 24-h samples of network activity can only provide a brief window into a lifetime of brain function. For instance, although we did not observe seizures in transgenic mice with tau knocked out, we cannot conclusively state that A53T/mTau^−/−^ mice do not have seizures unless they undergo constant surveillance. Future studies involving these models must focus on gathering more frequent, repeated measurements in a cohort that has been consistently tracked over time in order to precisely establish how these network deficits progress with age.

There is a growing body of knowledge linking network hyperactivity with exacerbated cognitive impairment in a variety of neurodegenerative paradigms. Though the work presented in the current study characterizes a transgenic model that represents only a fraction of α-synucleinopathies, the observation of similar phenomena in different models may have more broad translational significance. The utility of an anti-epileptic focus in treating dementia may extend beyond the realm of AD, and work expanding on our findings may pave the way for effective treatment in the early stages of α-synucleinopathies.

## Data Availability Statement

The datasets generated for this study are available on request to the corresponding author.

## Ethics Statement

The animal study was reviewed and approved by the Institutional Animal Care and Use Committee of the University of Minnesota.

## Author Contributions

SV and ML provided animals. JC and SV maintained animal colonies. SP, JC, and AF carried out electrode implantation and video-EEG/EMG recordings. SP and AF processed data and performed statistical analysis. ML and KV created the study concept and experimental plan and oversaw the study. All authors reviewed and interpreted data. SP prepared the figures. SP, AF, and KV drafted the manuscript. All authors contributed to the article and approved the submitted version.

## Conflict of Interest

The authors declare that the research was conducted in the absence of any commercial or financial relationships that could be construed as a potential conflict of interest.
