# Supplementary material for: Ablating Tau Reduces Hyperexcitability and Moderates Electroencephalographic Slowing in Transgenic Mice Expressing A53T Human α-Synuclein
Source: Front Neurol. 2020 Jun 19;11:563. doi: 10.3389/fneur.2020.00563 (PMC7316964; doi:10.3389/fneur.2020.00563)
Supplement: Supplementary file 2 [file Image_1.PDF]

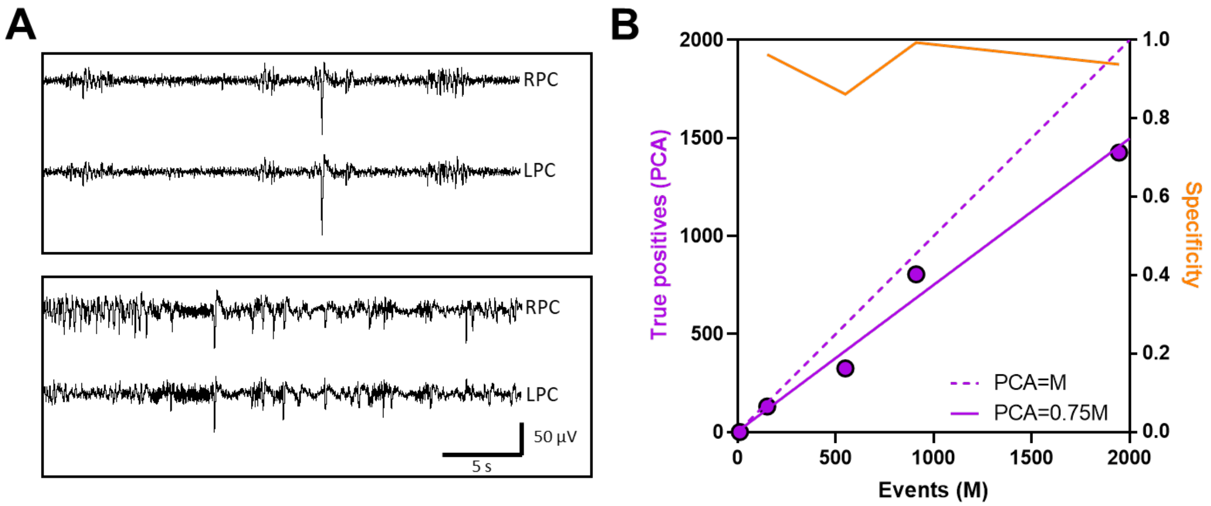

Supplementary Figure 1. **Semi-automated detection of large epileptiform events by PCA-K means clustering.** (**A**) Representative traces during behavior that tended to cause artifacts, including eating (top panel) and repetitive grooming/scratching (bottom panel). Waveforms from these behaviors occasionally resembled epileptiform events, but were typically larger in amplitude and had wider initial deflections (>100 ms). Questionable templates corresponding to artifact waveforms were evaluated and excluded by manual review of synchronized video. RPC, right parietal cortex; LPC, left parietal cortex. (**B**) Comparison of iterative PCA-K means clustering to manual counts of epileptiform events (M) from five 24-hour recordings revealed that the semi-automated method had a sensitivity (true positives detected by PCA out of all manually marked events) of approximately 75% (solid purple regression) in a wide range of excitability states (dashed purple line represents a sensitivity of 100%). The average specificity (number of PCA-marked events that were true positives) of this method in the four non-zero evaluated recordings was approximately 93% (orange solid line). The recording with zero PCA-marked events over 24 hours was found to have 11 false negatives (~0.5 events per hour). Recordings were evaluated from three A53T mice and two A53T/mTau$^{-/-}$ mice.
